# A Review of Febrile Seizures: Recent Advances in Understanding of Febrile Seizure Pathophysiology and Commonly Implicated Viral Triggers

**DOI:** 10.3389/fped.2021.801321

**Published:** 2022-01-13

**Authors:** Rana Sawires, Jim Buttery, Michael Fahey

**Affiliations:** ^1^Department of Paediatrics, Faculty of Medicine, Nursing and Health Sciences, Monash University, Clayton, VIC, Australia; ^2^Murdoch Children's Research Institute, Royal Children's Hospital, Parkville, VIC, Australia; ^3^Child Health Informatics, Department of Paediatrics, University of Melbourne, Parkville, VIC, Australia; ^4^Department of Neurology, Monash Children's Hospital, Clayton, VIC, Australia; ^5^Neurogenetics Department, Monash Paediatrics, Monash University, Clayton, VIC, Australia

**Keywords:** febrile seizure (FS), infectious diseases, neurology, viruses, paediatrics

## Abstract

Febrile seizures are one of the commonest presentations in young children, with a 2–5% incidence in Western countries. Though they are generally benign, with rare long-term sequelae, there is much to be learned about their pathophysiology and risk factors. Febrile seizures are propagated by a variety of genetic and environmental factors, including viruses and vaccines. These factors must be taken into consideration by a clinician aiming to assess, diagnose and treat a child presenting with fevers and seizures, as well as to explain the sequelae of the febrile seizures to the concerned parents of the child. Our article provides an overview of this common childhood condition, outlining both the underlying mechanisms and the appropriate clinical approach to a child presenting with febrile seizures.

## Introduction

Febrile seizures are the most common seizure of childhood (1). They are seizure events occurring in young febrile children, where the fever is not due to infection of the central nervous system (CNS) (1). The peak incidence in children is between 12 months and 18 months of age (2, 3).

This article will provide an overview of febrile seizure epidemiology and risk factors, clinical presentations, and current management, as well as triggers commonly implicated in febrile seizures. There have been surprisingly few similar overviews of febrile seizures, the most recent one dating to 2002 (4). Recent advances in our understanding of the pathophysiology of fever, inflammatory cascades, underlying genetic causes and the developing brain will also be discussed.

## Definitions And Classification

Febrile seizures are seizure episodes that occur in the presence of a fever (>38.0°C/100.4°F), usually in the context of a viral infection, and commonly occur in children between 6 months and 5 years old. This definition excludes seizures occurring in the presence of an underlying CNS infection or metabolic disturbance (1). Children with previous afebrile seizures are excluded from the group of children with febrile seizures as the febrile illness is perceived as a trigger of a pre-existing predisposition to epilepsy (5).

Febrile seizures are classified as either simple or complex based on their clinical features. Simple febrile seizures are single, generalised convulsions lasting <15 min. Complex febrile seizures present with focal features, occur as clusters of episodes during the same 24-h period (multiple seizures) or are prolonged with a duration longer than 15 min (1). Approximately 20–35% of febrile seizures are complex (2, 6).

Febrile status epilepticus (FSE) is variably defined as seizures lasting longer than 30 min (7). FSE accounts for 25–52% of all status epilepticus in children, although it is a small fraction of febrile seizure occurrences (7, 8). Children who have FSE have a greater risk of future adverse events, with up to 41% subsequently having febrile seizure recurrence (7). Children with underlying neurological abnormalities are at a greater risk of having FSE, comprising nearly a fifth of all children who experience FSE (7). Nevertheless, the overall mortality and morbidity of febrile status epilepticus are low (1, 7).

## Epidemiology

Febrile seizures are the most common cause of seizures in childhood, with a 2–5% incidence in European and American children (1). However, a higher incidence has been described in Japan (7–10%) (9) and Guam (14%) (10). Incidence appears to be unaffected by gender (3). While most febrile seizures occur between 6 months and 5 years of age, there are reports of first febrile seizures in children up to 7 years old (2), and as young as 3 months old (3). The highest incidence is in children aged 12–18 months old (3). Febrile seizures are most commonly reported in winter (11), corresponding with peaks in the occurrence of febrile illnesses in young children (12).

## Risk Factors Introduction

### Risk Factors for the First Febrile Seizure

Viral infections are the most common cause of the febrile illnesses associated with febrile seizure, being detected in up to 82% of children with febrile seizures (13). Some viral infections are associated with a higher incidence of febrile seizures (13). Some vaccinations are also a known risk factor for febrile seizures in children with the risk period following vaccination varying between vaccines (6). However, the risk is lower than that posed by the wild type viral infections the vaccines prevent (14). Fevers above 38°C and shorter fever durations increase the likelihood of febrile seizures (15, 16).

A positive family history of febrile seizures or epilepsy increases the risk of febrile seizures and has been described in 25–40% of children presenting with febrile seizures (17). Generalised epilepsy with febrile seizures plus (GEFS+) is a familial epilepsy syndrome which can manifest as febrile seizures in individuals (18). This syndrome may account for part of the observed familial predisposition.

There is a higher prevalence of febrile seizures in children with underlying neurological deficits, such as cerebral palsy or neurodevelopmental delay (19, 20). Neonatal discharge at 28 days of age or later (20), and low serum zinc and iron levels have also been associated with increased risk of febrile seizures (21, 22). Some environmental risk factors have been associated with increased febrile seizure incidence, including maternal smoking and stress (5, 20). Ultimately, the risk for febrile seizures increases exponentially, with a Canadian case-control study demonstrating that the risk of febrile seizure incidence increases to 28% in children with two risk factors (23). Table 1 provides a summary of febrile seizure risk factors.

**Table 1:** Risk factors of first febrile seizure.

**Risk factor**	**Odds ratio (95% CI)**
Family history of febrile seizures^*^	4.5 (2.09–9.83) (23)
Family history of afebrile seizures and epilepsy	2.6 (0.5–14.3) (15)
Developmental delay^*^	4.9 (1.55–15.5) (23)
Viral infection^*^	3.5 (2.2–5.6) for respiratory or enteric virus detection in children with febrile seizures compared to healthy controls (25).
High fever temperature^*^	1.8 (1.3–2.5) for every degree above 101°F (15).
Maternal smoking	3.0 (1.0–9.0) if child exposed to any perinatal smoking (15).
Neonatal discharge >28 days	5.6 OR (1.55–20.5) (23)
Low serum zinc^*^	1.5 (1.1–2.3) (21)
Low serum iron^*^	1.84 (1.02–3.31) (22)

* *Denotes statistically significant risk factors*.

### Risk Factors for Febrile Seizure Recurrence

Febrile seizure recurrence occurs in 30–50% of children following the first febrile seizure. Each additional febrile seizure increases the risk of further recurrence, suggesting that experiencing febrile seizures leads to a lower threshold for future seizures (24). This is highlighted by the fact that lower fever temperatures are associated with a greater risk of recurrence of febrile seizures (24). Table 2 describes other risk factors which contribute to febrile seizure recurrence. In general, there is an individual seizure threshold which is lower in younger children and in those with a family history of febrile seizures. The risk of febrile seizure recurrence increases when more than one risk factor is present, but the exact effect is uncertain (19, 20, 28).

**Table 2:** Risk factors for febrile seizure recurrence.

**Variables**	**Risk factor**	**Risk ratio**	**95% CI**
Individual child	Younger than 12 months at first febrile seizure^*^	2.40 (26)	1.42–4.06
	Family history of febrile seizures^*^	1.89 (27)	1.23–2.90
	History of febrile seizures^*^	1.98 after one previous recurrence (24).	1.72–2.27
		2.59 after two or more recurrences (24).	2.20–3.04
Initial febrile seizure	Complex febrile seizure	1.08 (27)	0.71–1.64
	Febrile status epilepticus	0.94 (27)	0.38–1.83
Fever	Fever <40°C^*^	1.54 (24)	1.25–1.89
	Short fever duration (seizure occurrence within 1 h of fever onset)^*^	4.62 (26)	1.35–5.80

* *Denotes statistically significant risk factors*.

### Risk Factors for Other Febrile Seizure Sequelae

Most seizures resolve spontaneously, and children recover within 24 h with minimal sequelae, particularly in simple febrile seizures (19).

However, a child who has had a febrile seizure has a greater risk of experiencing afebrile seizures or epilepsy subsequently. Younger children, those with a family history of seizures or epilepsy and those who have complex seizures, have the greatest likelihood of developing further afebrile seizures or epilepsy (2). Nevertheless, it is important to recognise that the exact relationship between febrile seizures and epilepsy is still uncertain. A correlation between the two may be due to an underlying brain abnormality that predisposes a child to both febrile seizures and epilepsy, or febrile seizures may prime the developing brain, making it more susceptible to later life epilepsy. Factors which have been identified to increase the incidence of afebrile seizures and epilepsy among children with a history of febrile seizures have been summarised in Table 3.

**Table 3:** Risk factors predisposing to afebrile seizures and epilepsy following febrile seizure incidence.

**Afebrile seizures**
**•Family history of epilepsy** **•Abnormal neurological status** **•Complex febrile seizures** **•Prolonged seizures and FSE** **•Febrile seizure recurrence**
**Epilepsy**
**•Prolonged febrile or FSE** **•Febrile seizure recurrence** **•Family history of epilepsy** **•Low APGAR scores at 5 min** **•Personal history of cerbral palsy** **•Pre-existing neurological abnormality**

Simple febrile seizures have consistently found to be benign, and most children have normal growth and development (1, 19). Although concern about long-term effects is a cause of parental distress (29), serious outcomes are rare; intellectual or behavioural impact are not reported (30), and there is no increase in mortality compared to the general population 2 years after the seizure (31). Nevertheless, family disruption and parental anxiety are observed in the 2 weeks following an initial febrile seizure (32).

## The Role Of Viruses And Vaccinations

### Viruses

Febrile seizures have been more strongly associated with certain viruses than others (6, 13, 25, 33). Key viruses known to contribute to febrile seizures in young children are shown in Table 4. Viral infections are documented in up to 80% of febrile seizures (35), a figure that is comparable to the rate of viral infections in febrile children without seizures (36). The fall/winter seasonality in numerous studies (11, 12), supports the association of febrile seizures with Upper Respiratory Tract Infections (URTIs) and their common causative viruses, including influenza viruses and respiratory syncytial virus (RSV) (3, 34). Peaks of febrile seizure incidence in summer months correlate with increased gastroenteritis diagnoses in children in summer, including enteroviruses (34). Nevertheless, many other viruses have been associated with febrile seizures (Table 3), but do not contribute to seasonal trends as clearly. Human herpesevirus-6 (HHV-6), also known as roseola infantum or sixth disease, is an important virus historically implicated in febrile seizures (6). While children with febrile seizures in the setting of HHV-6 infection may experience respiratory or gastrointestinal symptoms, exanthema is a more common finding in these children than in a group of all children with febrile seizures (37).

**Table 4:** Viruses associated with febrile seizures, peak seasons, and prevalence in children with febrile seizures.

**Virus**		**Seasonal peaks**	**% of all febrile seizures**
Influenza A and B		Winter	15–50% (25, 34)
Respiratory syncytial virus		Winter	9% (6)
Adenovirus		None	11–21% (6, 13)
Human metapneumovirus		Winter	2–3% (6, 25)
Parainfluenza	1	Biannual, Summer-Autumn	10–18% (23, 26)
	2	Biannual, Winter	
	3	Spring	
	4a and 4b	Summer-Autumn	
Rhinovirus		Spring-Early Autumn	14–22% (6, 25)
Rotavirus		Winter	1.3% (35)
Enterovirus		Summer	20–38.9% (6, 34)
Human herpes virus-6		None	20% (6)

### Vaccinations

Febrile reactions following vaccinations are common in young children. Most childhood vaccinations occur in this age group, and 11% of febrile seizure presentations may occur within 2 weeks post-vaccinations (6). Table 5 outlines the known febrile seizure risk in relation to common childhood vaccinations. The role of vaccinations highlights the involvement of an infective agent in febrile seizures is two-fold. Firstly, some vaccinations reduce febrile seizure risk by preventing infection, thus highlighting the role of certain viruses in febrile seizures. Secondly, although some vaccinations both pose an increased chance of febrile seizure incidence in a short period of time following vaccination, they ultimately prevent infection by common viruses which cause febrile seizures and reduce the risk overall. These associations underline the importance of viral infections in febrile seizures.

**Table 5:** The association between vaccines and febrile seizures.

**Vaccine**	**Febrile seizure risk period**	**Association with febrile seizures**
Influenza	Days 1–3 post-vaccination	2.0 RR^*^ (95% CI 1.15–3.51) (14)
		No increase with current Influenza vaccine (38)
MMR	Days 5–12 post-vaccination	2.75 RR^*^ (95% CI 2.55–2.97)−2.83 RR (95% CI 1.44–5.55) (39, 40)
MMRV	Days 5–12 post-vaccination	1.08 RR^*^ (95% CI 0.55–2.13) (41) (First dose only)
DTP	Days 0–2 post-vaccination	No increased risk (42)
		5.70 RR^*^ (95% CI 1.98–16.42) (39)
DTaP-IPV-Hib	Days 1–2 post-vaccination	3.94 RR^*^ (95% CI 2.18–7.10)−6.02 RR^*^ (95% CI 2.86–12.65) (43)
Rotavirus	None	0.79 RR^*^ (95% CI, 0.71–0.88) for all-seizure incidence (44).

* *RR, Risk Ratio*.

## Pathophysiology

Seizures occur due to synchronised, prolonged, and unchecked activation of clusters of neurons, and arise out of a mismatch in excitatory and inhibitory activity in the brain (45). The exact pathophysiology of febrile seizures is unknown. However, it is assumed that a combination of genetic predisposition and environmental factors- the fever and its cause- trigger the event.

### Inherited Predisposition

#### Hippocampal Atrophy

Associations between reduced hippocampal and amygdala size, and families with a greater prevalence of febrile seizures and other seizure syndromes have been described. One exploratory study which compared the age and sex of matched controls experiencing febrile seizures showed that febrile seizures were more prevalent in families who had relatively smaller hippocampal and amygdala volume, marked asymmetry in these structures as well as hippocampal malformations (46). These findings suggest that structural malformations of the hippocampi underly the mechanism of febrile seizures (rather than being sequelae of febrile seizures). The FEBSTAT study of 3 cohorts of US children with febrile seizures showed that those with FSE were more likely to have increased hippocampal T2 signal on MRI, compared with children who did not experience prolonged febrile seizures (47). This increased signal suggests hippocampal abnormalities are more common in children who experience more severe febrile seizures. However, it is unknown whether the hippocampal abnormalities preceded episodes of FSE or were a consequence of the prolonged seizures.

#### Genetic Susceptibility

Given the familial risk factors observed in febrile seizures, it is unsurprising that genetic associations with febrile seizures and other seizure syndromes at both the receptor and ion channel level have been identified (45). Two key mutations are described below.

##### Voltage-Gated Sodium Ion Channels

Voltage-gated sodium ion channels play an integral role in the propagation of an action potential in neurons. Variations in genes coding for the sodium channel protein have been found, confirmed by the discovery of many sodium channel subtypes (48). In studies of families GEFS+, mutations of the gene SCN1B have been found in all individuals who present with seizures, including febrile seizures (49). A recent study also showed mutations in the gene SCN1A in association with two families with GEFS+ syndromes (50). Other mutations of the SCN protein have been identified and are shown to be present in phenotypically similar families (51). Nevertheless, GEFS+ is an uncommon cause of febrile seizures (18), thus it is difficult to determine the exact role played by mutations in sodium channel proteins.

##### Hyperpolarisation Activated Cyclic Nucleotide-Gated Channels

Hyperpolarisation activated cyclic nucleotide-gated (HCN) channels are key channels in seizure development, facilitating neural excitability (52). Mutations to hyperpolarisation genes activated cyclic nucleotide-gated (HCN) channels have been found in patients with seizures and epilepsy, underpinning their role in neuronal excitability. HCN1 mutations have been associated with a wide spectrum of seizure disorders, including febrile seizures (53). One heritable mutation of HCN2 described in a 2013 Japanese study made HCN channels more readily activated in response to higher temperatures, promoting seizure activity (54). Further, specific HCN2 channel mutations have been found in association with febrile seizure and GEFS+ patients, but not idiopathic generalised epilepsy patients (55), implying a specific role for HCN2 channels in seizures precipitated by fevers.

Apart from SCN and HCN channel mutations, other genetic mutations have been implicated in febrile seizures (56–58). Mutations are not always identified in children with febrile seizures (58), which may indicate that other undiscovered genetic changes contribute to febrile seizure propagation. Regardless, it is also important to note that while these ion channel mutations play a role in febrile seizures, they are shown to have low penetrance (51, 53). Thus, a second factor may be necessary for seizure propagation.

### Fevers and Seizure Induction

#### Neuron Excitation

Recent studies have proposed that prenatal and early postnatal stressors may influence mechanisms that lead to limbic epileptogenesis, by altering the developing brain's neuroplasticity (59). Early-life trauma (such as maternal infection, prenatal maternal or environmental stress, perinatal hypoxic-ischaemic injury or postnatal infection, seizure or traumatic brain injury) is thought to lead to modification of circuit excitability by recruiting astrocytes and microglia at the site of insult. Following this reduction in seizure threshold in the developing brain, a “second-hit” (e.g., a fever) may be sufficient to trigger seizure activity (5).

Increasing the temperature of the brain has been suggested to increase neuronal firing, which in turn increases the likelihood of synchronised neuronal activity that leads to seizure induction (60). Specifically, hyperthermia alone can increase the excitability of pyramidal and dentate granule cells, as well as that of inhibitory interneurons (61). Febrile seizures may also lead to ectopic granule cells, which are more aberrant and tortuous, and render the brain hyperexcitable. This could explain the increased risk of febrile seizure recurrence after a first seizure (62). These mechanisms potentiate seizure activity as increased excitability reduces the seizure threshold, and the increased inhibition of the interneurons augments the synchronicity of cells.

#### Fever and Immune Pathway Activation

**Figure 1:**
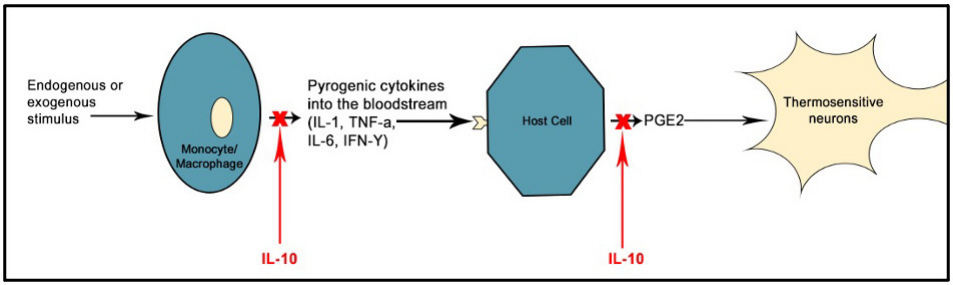
Simplified model of the febrile response and key pro- and anti-inflammatory cytokines. Adapted from Mackowiak et al. (63).

In addition to affecting the neural pathway, fevers may trigger febrile seizures via the inflammatory pathway. IL-1α and IL-1β, TNF-α, IL-6, and Interferon (IFN) are all cytokines of the pyrogenic pathway, with IL-10 is an anti-inflammatory cytokine produced in response to IL-1β, IL-6, and TNF-α (Figure 1) (63). Both IL-1β and IL-10 are elevated in febrile seizures (64, 65). A rodent study comparing rats with and without febrile seizures demonstrated increased IL-1β levels specifically in the hippocampus at the onset of a febrile seizure, and these levels were maintained throughout (66). Antipyretics that inhibit prostaglandins do not reduce the duration or recurrence of febrile seizures (67). This indicates that prostaglandins [produced due to cytokine release in the febrile response (63), as shown in Figure 1] do not initiate seizures in febrile illnesses.

Most febrile illnesses do not result in seizures. Different viral infections are associated with increases in specific inflammatory cytokines (Table 6). Studies have shown more frequent genetic mutations in IL-1α and IL-1β genes in patients with temporal lobe epilepsy, and IL-1β in patients with febrile seizures compared with healthy controls (68, 69). Such mutations may lead to the production of seizure-promoting variations in these inflammatory cytokines. Ultimately, febrile seizures are likely multi-factorial and a pre-existing state of neuronal hyperexcitability may be required for inflammatory cytokines to propagate seizures.

**Table 6:** Cytokines and their role in febrile seizure induction.

**Cytokine**	**Role**	**Association**	**Key viruses**
IL-1β	Pro-inflammatory	•Increased up to 5 h following febrile seizures (64).	HHV-6 (6)
IL-6	Pro-inflammatory	•Up to 98% increased production in cells of children with a history of febrile seizures (64). •Level of IL-6 may indicate the severity of the disease (17).	Influenza (65)
TNF-α	Pro-inflammatory	•Found in 76% of patients with influenza-induced encephalopathy and febrile seizures due to influenza (65).	Influenza (65)
IL-10	Anti-inflammatory	•Increased in children with a febrile seizure history (64). •Increases mortality (65). •Level of IL-6 may indicate the severity of the disease (17).	Influenza (65)

## Management

### Investigation

A presentation of fevers with seizures has a wide range of differentials and investigations should aim to determine the cause (70). For simple febrile seizures, no tests should be performed unless other symptoms indicate it. Complex febrile seizures are often a diagnosis of exclusion following an appropriate work up.

Full blood count, serum electrolytes, calcium, magnesium, phosphorous and blood glucose levels are not routinely recommended for evaluating febrile seizures, as they will not change the management course. These should only be performed if other clinical features indicate the need for these investigations, such as prolonged post-ictal drowsiness or suspicion of bacteraemia (1, 19, 70).

In young children, seizures are a common presentation of meningitis; thus, it is essential to exclude this diagnosis. While lumbar punctures are not clinically indicated for most children presenting with seizures, the presence of meningism or a history suggestive of meningitis or intracranial infection are indications for a lumbar puncture. In children <18 months of age, clinical signs of meningism are unreliable (70). In infants 6–12 months old presenting with fever and seizure, a lumbar puncture is indicated if there have been no Hib or pneumococcal vaccinations (or if the history of these vaccinations is unknown), and to rule out meningitis or other intracranial infection if there is clinical suspicion (1, 70). A lumbar puncture may be performed for children 13–18 months old if there is sufficient clinical suspicion for meningitis, or in a child with FSE (1, 19, 70).

Electroencephalograms (EEGs) have been variably recommended for investigating febrile seizures (71). However, these add little to the management of a child with febrile seizures or FSE. While EEGs may show slow or focal abnormalities in 34% of children with febrile status epilepticus, the absence of epileptiform activity does not exclude seizures. Therefore, it is not considered useful for routine diagnosis (1). Neuroimaging is not recommended in febrile seizures for assessing the risk of recurrence or long-term neurological harm and is rarely needed for simple febrile seizures (70, 72). Computerised tomography (CT) and magnetic resonance imaging (MRI) are only indicated to exclude other pathology as suspected, including focal lesions, structural defects or severe head injury (19). If other diagnoses are excluded, CT and MRI do not alter the management plan in a diagnosis of febrile seizures.

### Treatment and Prevention

Randomised control trials have shown that benzodiazepines including midazolam, diazepam and lorazepam have a therapeutic effect in children with seizures. Nevertheless, the efficacy of these drugs in children with febrile seizures is uncertain (73, 74). It is accepted that early intervention is unnecessary for simple febrile seizures. However, rectal or intravenous diazepam or intravenous lorazepam should be administered for any child with FSE (72). Some drugs may be effective at reducing the risk of subsequent recurrence for complex and simple febrile seizures, including phenobarbital, primidone, valproate, and intermittent diazepam (67, 75). Nevertheless, the severity and incidence of adverse effects, including the risk of respiratory depression (74), associated with these drugs frequently outweigh potential benefits, so they are infrequently used (75). Despite the established link between the febrile immune response and febrile seizures (64, 65), antipyretics benefits are generally limited to providing comfort to the child and do not affect the risk of future recurrence or severity (19, 67). Only one randomised control study in Japan has shown that rectal acetaminophen may reduce the risk of febrile seizure recurrence within the same febrile episode (76).

## Conclusion

Febrile seizures are common childhood illnesses and the great majority resolve spontaneously with a typical neurodevelopmental outcome. Febrile seizure incidence appears to be the result of the interplay of genetic and environmental factors, including common childhood viral infections. Although benign and requiring minimal management, the prevalence of febrile seizures and the possibility of long-term sequelae such as recurrence, afebrile seizures and epilepsy make them clinically important presentations in children.

## Author Contributions

RS performed the literature review for this article and was a major contributor in writing the manuscript. All authors read and approved the final manuscript.

## Conflict of Interest

The authors declare that the research was conducted in the absence of any commercial or financial relationships that could be construed as a potential conflict of interest.

## Publisher's Note

All claims expressed in this article are solely those of the authors and do not necessarily represent those of their affiliated organizations, or those of the publisher, the editors and the reviewers. Any product that may be evaluated in this article, or claim that may be made by its manufacturer, is not guaranteed or endorsed by the publisher.
